# Potential of Liver Serum Enzymes and SUVmax in Primary Tumors as Predictive Biomarkers With Correlational Evidence

**DOI:** 10.7759/cureus.58532

**Published:** 2024-04-18

**Authors:** Sashikanta Swain, Abhijit Sahu, Parneet Singh, Sipra Rout, Girish K Parida, Pravash R Mishra, Kanhayalal Agarwal

**Affiliations:** 1 Anatomy, All India Institute of Medical Sciences, Bhubaneswar, Bhubaneswar, IND; 2 Nuclear Medicine, All India Institute of Medical Sciences, Bhubaneswar, Bhubaneswar, IND

**Keywords:** lymphoma, cancer, suv max, liver enzymes, pet/ct

## Abstract

Introduction

Cancer exerts a substantial influence on the body's metabolism through varied mechanisms, instigating a metabolic reprogramming that maintains the unchecked growth and survival of cancer cells, consequently perturbing diverse metabolic parameters. The introduction of positron emission tomography-computed tomography (PET/CT), delivering detailed insights into both metabolic and morphological aspects, has brought about a revolutionary shift in modern cancer detection. Exploring the potential connection between PET-CT metabolic features and the metabolic parameters of liver enzymes in an individual can unveil novel avenues for cancer diagnosis and prognosis.

Materials and methods

This study conducted a retrospective analysis of patient records from our institution, covering the period from January 2021 to September 2023, focusing on individuals with various malignancies. The data included information on gender, age, clinical history, and liver serum parameters, which were compiled into tables. Additionally, inflammatory indicators such as ALT (alanine transaminase), ALP (alkaline phosphatase), total protein (TP), ALT/AST ratio, and SUVmax were collected and plotted. The study used Pearson correlation analysis to assess the relationship between each inflammatory variable and SUV (max) as determined by PET-CT.

Results

In breast cancer, there was a statistically significant positive correlation (R2=0.0651) between serum ALP levels and SUVmax as determined by regression analysis. Hodgkin lymphoma, on the other hand, showed a statistically significant negative correlation between the ALT-to-AST ratio (ALT/AST) and SUVmax (r = -0.45, R2 = 0.204). In non-Hodgkin lymphoma patients, total protein (TP) was negatively correlated with SUVmax (R2=-0.081, r= -0.28), while in lung cancer patients, there was a significant positive correlation with regression correlation coefficients (R2 = 0.026, 0.024, 0.024, and 0.018 for ALT/AST, TP, ALP, albumin, and ALT, respectively).

Conclusion

Aligning with these results, it can be a recent addition to acknowledge that both the tumor metabolic parameter (SUVmax) and the levels of liver serum enzymes exhibit a potential for predicting patient prognosis in various cancers.

## Introduction

Cancer profoundly influences the body's metabolism through various mechanisms, leading to metabolic reprogramming that supports cancer cells' uncontrolled growth and survival through the Warburg effect, increased glucose consumption, altering amino acid and lipid metabolism, and mitochondrial dysfunction [1]. In the 21st century, cancer persists as the primary cause of mortality, still posing challenges for early detection. Various advanced technologies like liquid biopsy, artificial intelligence (AI) in imaging, genomic profiling, multi-parametric MRI (mpMRI), optical imaging techniques, cancer biomarkers, volatile organic compounds (VOCs) analysis, and advances in endoscopic technologies such as confocal laser endomicroscopy and molecular imaging are employed for the early detection of cancer [2-6]. Recently, positron emission tomography-computed tomography (PET-CT) has established itself as a crucial diagnostic tool for cancer, offering valuable insights into the physiological and anatomical features of malignancies [7]. When it comes to the first evaluation of cancer patients' grades and responses, PET-CT has a distinct advantage over morphological imaging since it combines functional and morphological data [7]. The widespread use of 18F-fluorodeoxyglucose (18F-FDG) PET/CT in oncological clinical practice has fundamentally altered the therapy of numerous malignancies. FDG PET/CT has been associated with widespread inflammation and the number of immune cell populations in the malignant microenvironment in those with cancer [8,9].

The dynamic interplay between liver biomarkers and cancer is a multifaceted landscape marked by evolving insights. Recent research has unearthed notable changes in the correlation between liver biomarkers and cancer, particularly in the context of breast, Hodgkin, non-Hodgkin, and lung cancers. There is mounting evidence that the cancer stage is associated with heightened levels of liver serum biomarkers [10-12]. The combined study of metabolic parameters and liver serum markers may considerably improve the early identification of many cancers [13-15]. The intricate interplay between population genetics and liver biomarkers is influenced by genetic variations present in diverse populations. Several studies have indicated that the levels of liver biomarkers, including enzymes like alanine aminotransferase (ALT) and aspartate aminotransferase (AST), can be influenced by genetic factors [16]. Polymorphisms in genes associated with liver function may contribute to variations in biomarker levels across populations [17]. For instance, genetic variants in genes encoding enzymes involved in drug metabolism or detoxification processes in the liver can impact the levels of specific biomarkers. Additionally, population-specific genetic variations may contribute to differences in susceptibility to liver diseases, affecting the baseline levels of biomarkers [16]. Understanding these genetic underpinnings is crucial for personalized medicine approaches, as individuals with specific genetic profiles may exhibit unique patterns in liver biomarkers. Understanding these alterations in liver biomarkers and varying degrees of FDG accumulations in the liver among individuals, we’d like to see whether there's an association between liver blood enzyme levels and primary tumor standard uptake value (SUV) on FDG-PET in the Indian population.

## Materials and methods

This study includes data from patient records who were diagnosed with diverse malignancies and treated in our institute between January 2021 and September 2023. Before the patients underwent the PET/CT scan, the clinical information, including gender, age, clinical history, and liver serum parameters (e.g., creatinine, alanine aminotransferase (ALT), aspartate aminotransferase (AST), alkaline phosphatase (ALP), bilirubin, albumin, and total protein (TP), was noted. We calculated the inflammatory indicators, i.e., absolute ALT, absolute ALP, absolute TP ALT/AST, and SUVmax. The following inclusion requirements were met while choosing the patient data: (a) cancer confirmed by biopsy specimens, (b) no prior therapy before PET/CT scanning, and (c) without any other illnesses that could significantly affect serum parameters. Before the commencement of the study, the ethics committee received approval from the institute for this research work. Based on the inclusion criteria, one hundred and one patients’ data were included out of 3500 in our research. Patients who had been pathologically determined to have breast, Hodgkin, non-Hodgkin, and lung cancer were included in the study based on the inclusion criteria.

Calculation of absolute value of liver parameters 

An advanced biochemical analyzer facility available at the institute was used to measure the liver serum enzymes included in our study, and the lab professionals double-verified the results. After getting these records of the patients, the absolute value of different serum enzyme values was calculated using the standard formula. The ALT-to-AST ratio was calculated by dividing the absolute ALT by the absolute AST value.

PET-/CT-derived parameters

**Figure 1:**
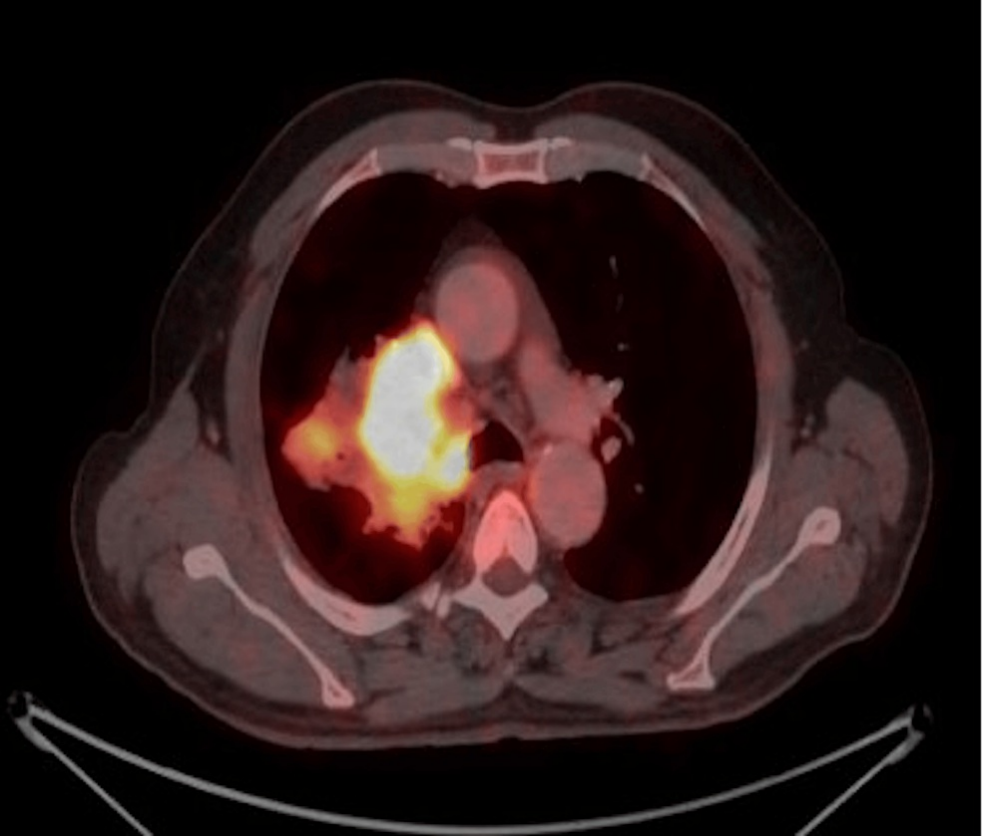
A PET CT image of a 59-year-old patient with a known case of small cell neuroendocrine carcinoma of the right lung. PET-CT was done for initial staging. Intensely FDG avid heterogeneously enhancing irregular lobulated hilar soft tissue mass with areas of necrosis is seen in the right lung, measuring 6.8 x 6.0 x 6.2 cm (AP x T x CC) with SUVmax 23.8.

All PET/CT scans were conducted using a GE Discovery MI DR PET/CT scanner (GE Medical Systems, Milwaukee, WI). A high-performance workstation and software (SyngoVia; Siemens, Erlangen, Germany) were used to analyze the FDG-PET/CT data. In the presence of the data analysis expert in oncological PET/CT, we visually assessed PET/CT images for elevated FDG uptake (Figure 1). The primary tumor with focally enhanced FDG uptake was regarded as positive for carcinoma compared to the background and liver FDG uptake. The formula was used to determine the lesions' maximum SUV values (SUVmax): SUVmax = tissue concentration (Bq/mL)/(injected dose (Bq)/body weight (kg).

Statistical analysis

The correlation between each inflammatory variable and SUV (max), as assessed through PET-CT, was examined using Pearson correlation analysis. This analysis explored the relationship between absolute ALT, absolute ALP, absolute TP, absolute albumin, and the ALT to AST ratio with PET/CT-SUV max for each case. A significance cut-off of P<0.05 was applied, and no adjustments for multiple testing were undertaken.

## Results

Demographic data on the various cancers included

**Figure 2:**
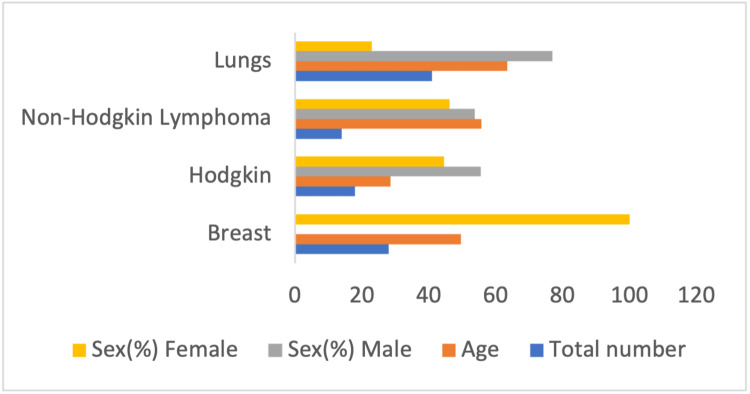
Showing the demographic data of the different cancer cases included.

In the present study, cancer types and the essential features of the individuals participating in our research are noted. Figure 2 shows the demographic data (age, sex) for each type of cancer. 

Correlation of serum liver enzyme levels and metabolic parameters in breast cancer

**Figure 3:**
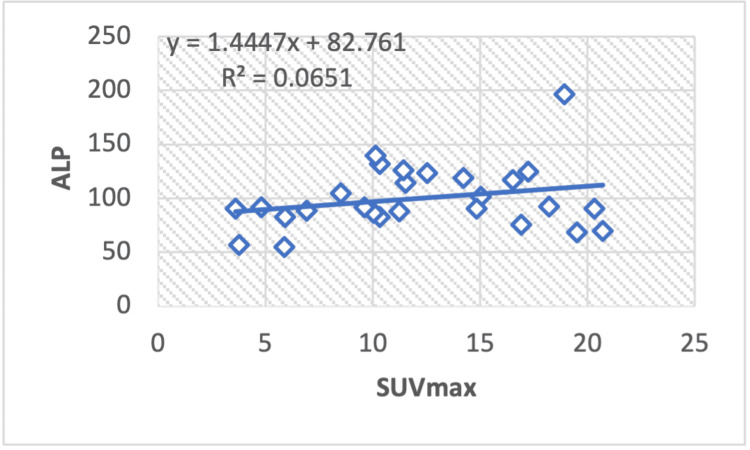
A regression analysis graph shows a correlation between the maximum standardized uptake value (SUV max) and the liver serum enzyme ALP (alkaline phosphatase) in breast cancer.

The regression analysis of serum ALP and the SUVmax among breast cancer patients reveals a statistically significant regression correlation value, R2=0.0651, with a calculated Pearson correlation coefficient of r = 0.25 (Figure 3).

Correlation of serum enzyme levels and metabolic parameters in Hodgkin lymphoma

**Figure 4:**
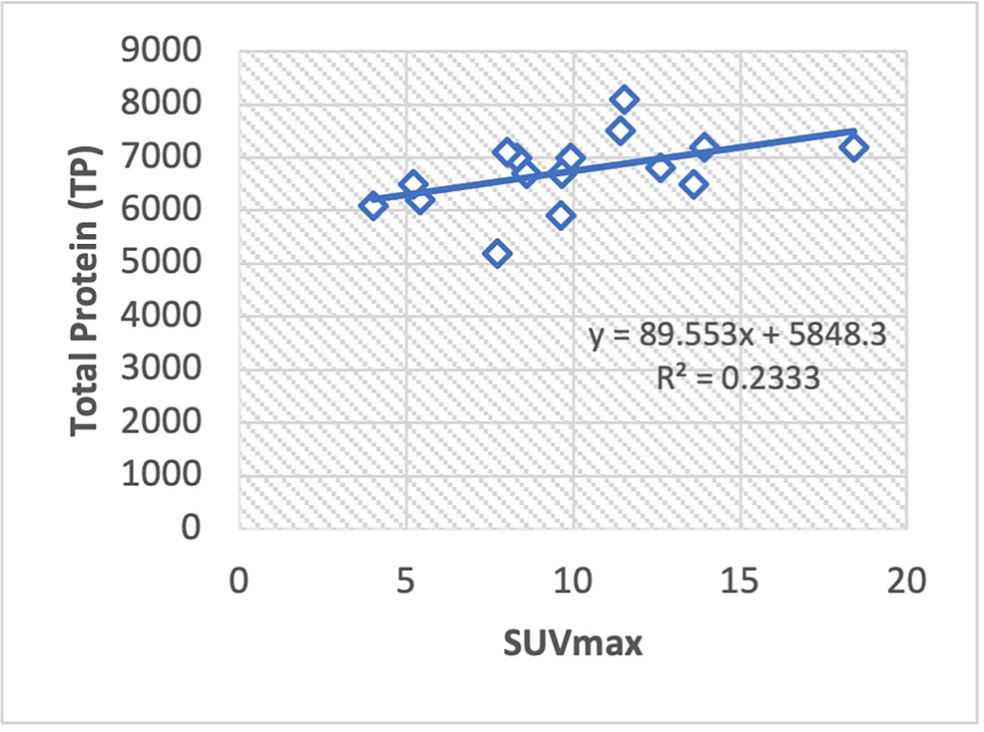
Regression analysis graph showing the correlation between maximum standardized uptake value (SUVmax) and liver serum enzyme TP (total protein) in Hodgkin's lymphoma.

**Figure 5:**
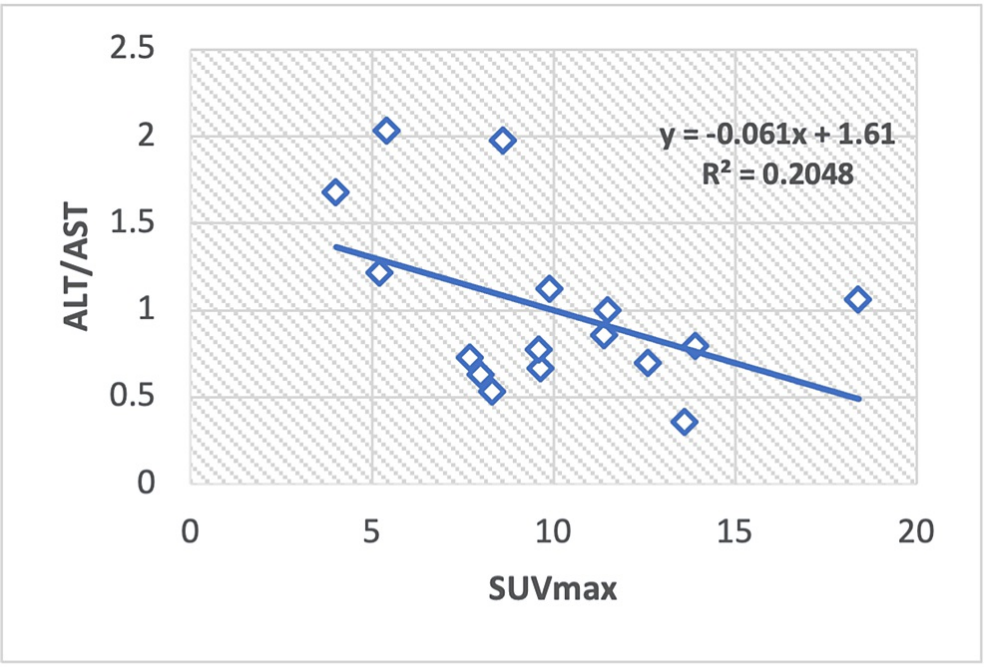
A regression analysis graph shows the correlation between the maximum standardized uptake value (SUVmax) and liver (ALT/AST) in patients with Hodgkin lymphoma. ALP: alkaline phosphatase; AST: aspartate aminotransferase

The regression analysis conducted on Hodgkin lymphoma patients reveals a correlation value between TP and the SUVmax value (R2 = 0.233), yielding calculated Pearson correlation coefficients of r = 0.48 (Figure 4). Conversely, a statistically significant negative correlation exists between the ALT to AST ratio (ALT/AST) and SUVmax value, resulting in calculated Pearson correlation coefficients of -0.45 and a regression value of R2 = 0.204 (Figure 5).

Correlation of serum enzyme levels and metabolic parameters in non-Hodgkin lymphoma

**Figure 6:**
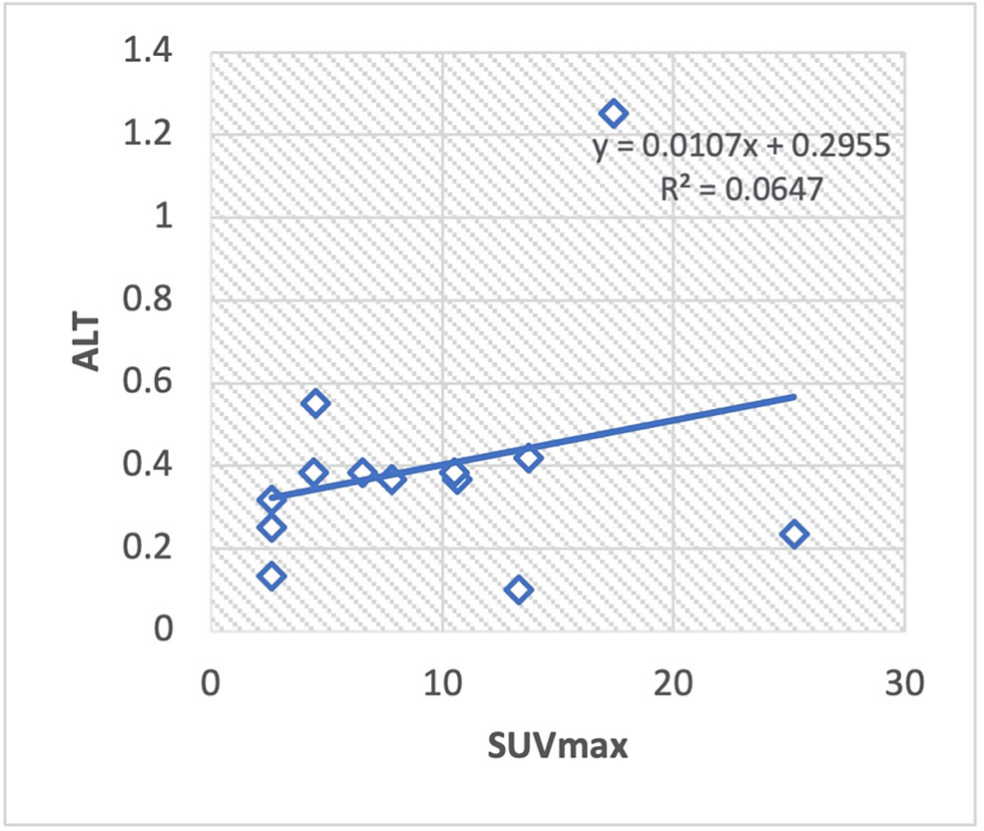
Regression analysis between maximum standardized uptake value (SUVmax) and liver serum enzyme ALT in non-Hodgkin lymphoma. ALT: Alanine transaminase

**Figure 7:**
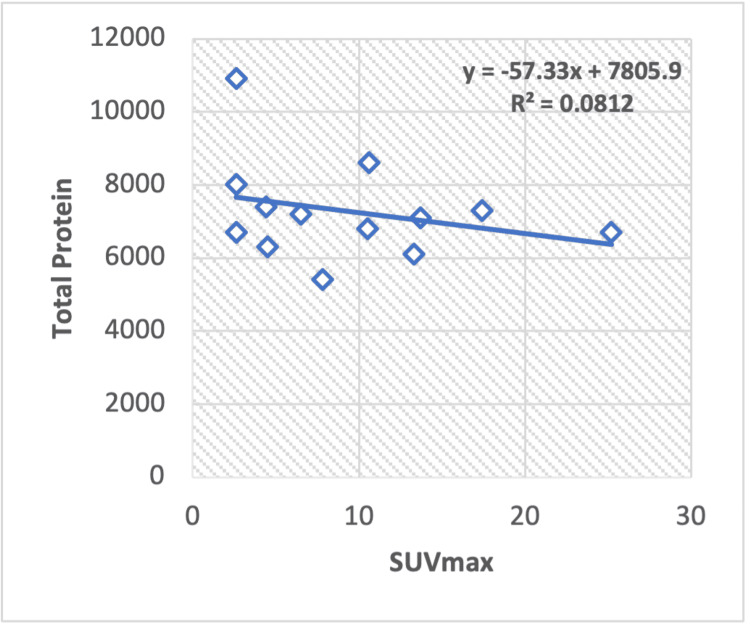
Regression analysis between maximum standardized uptake value (SUVmax) and liver serum TP in non-hodgkin lymphoma. TP: Total protein

Our regression analysis of liver serum enzymes ALT with the SUVmax of non-Hodgkin lymphoma patients shows a weak positive correlation (R2 = 0.0647) with a calculated Pearson correlation coefficient (r =0.25) (Figure 6). Similarly, TP negatively correlated with SUVmax (R2 = -0.081) with a calculated Pearson correlation coefficient of r = -0.28 (Figure 7). However, when examining other serum enzymes, such as albumin, in relation to the SUVmax value, no statistically significant correlation was observed.

Correlation of serum enzyme levels and metabolic parameter in lung cancer

**Figure 8:**
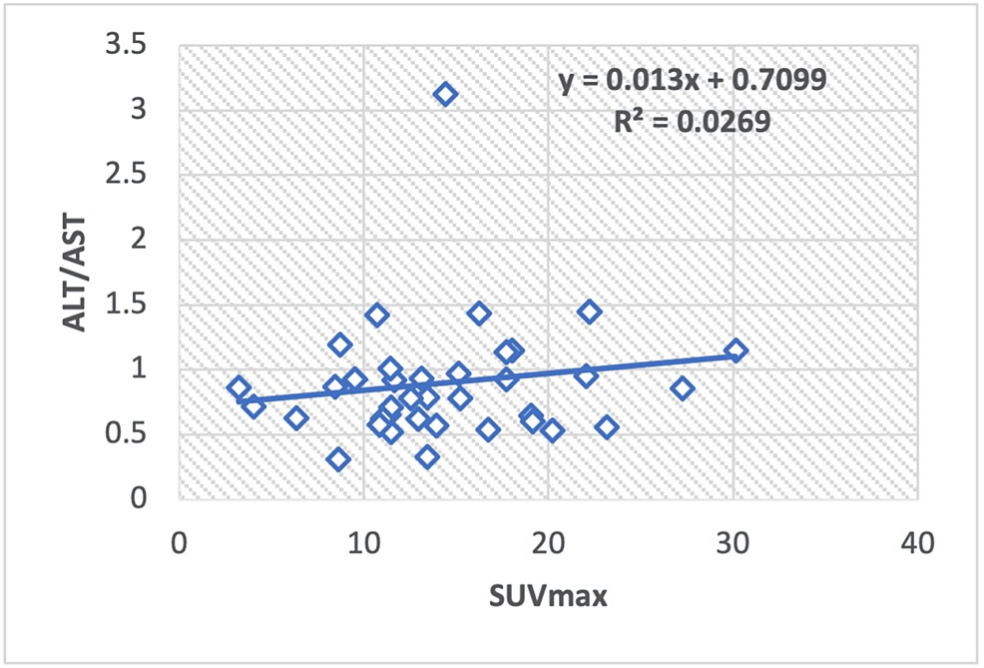
Regression analysis between maximum standardized uptake value (SUVmax) and liver serum enzyme ALT/AST in patients with lung cancer. ALT: Alanine transaminase AST: Aspartate aminotransferase

**Figure 9:**
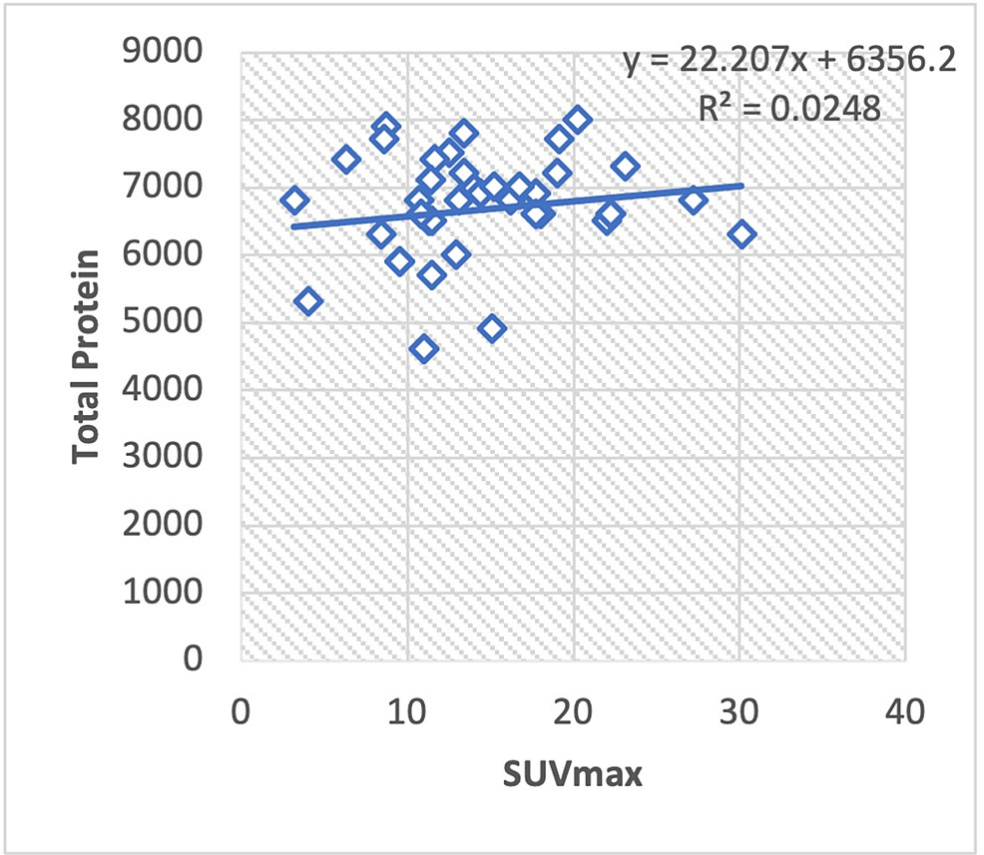
Regression analysis between the maximum standardized uptake value (SUVmax) on positron-emission tomography or computed tomography and liver serum enzyme TP in patients with lung cancer. TP: Total protein

**Figure 10:**
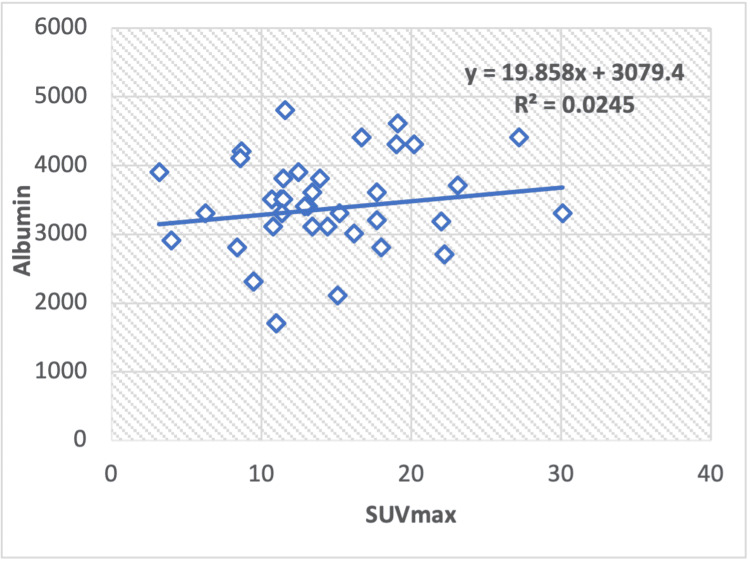
Regression analysis between maximum standardized uptake value (SUVmax) on positron-emission tomography or computed tomography and liver serum albumin in patients with lung cancer.

**Figure 11:**
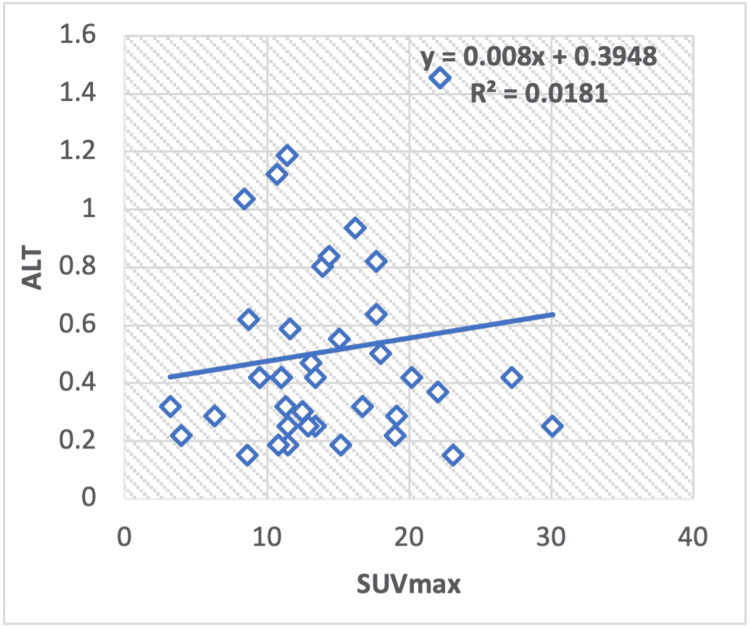
Regression analysis between maximum standardized uptake value (SUVmax) on positron-emission tomography or computed tomography and liver serum enzyme, ALT, in patients with lung cancer. ALT: Alanine transaminase

**Figure 12:**
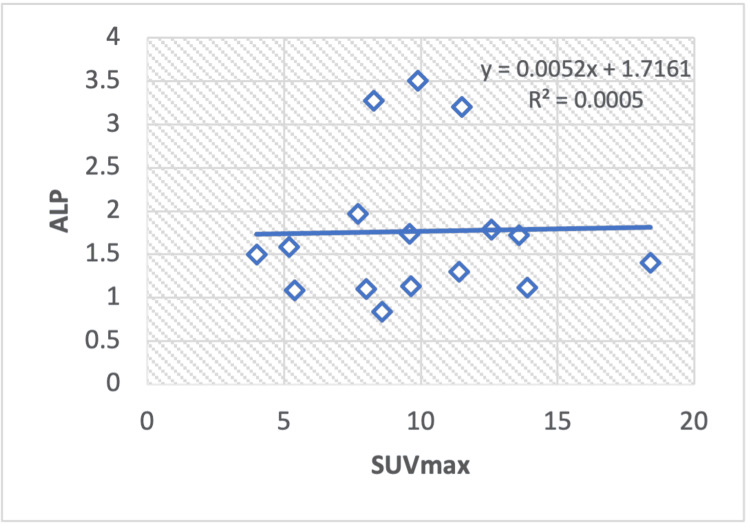
Regression analysis between the maximum standardized uptake value (SUVmax) on positron-emission tomography or computed tomography and liver serum enzyme ALP in patients with lung cancer. ALP: Alkaline phosphatase

In the regression analysis of liver serum enzymes, ALT/AST (Figure 8), TP (Figure 9), albumin (Figure 10), ALT (Figure 11), and ALP (Figure 12), with the SUV max of lung cancer patients, a significant positive correlation is observed, with calculated regression correlation coefficients of R2 = 0.026, 0.024, 0.024, and 0.018, respectively. A similar trend is noticed in the Pearson correlation (r = 0.16; ALT/AST, r = 0.15; TP, r = -0.01; ALP, r = 0.15; albumin, r = 0.13; ALT).

## Discussion

The present study aims to validate if any significant relationship exists between the liver serum enzymes (ALT/AST, TP, albumin, ALT, and ALP) and the maximum standardized uptake value (SUVmax). Research in this domain has illuminated the pivotal role of FDG-PET in detecting various types of cancer. Diverse physiological conditions influence the uptake of FDG, and there exists a potential for misinterpretation of PET scans, leading to false-positive or false-negative outcomes [18,19]. Consequently, attention has been directed towards investigating the physical variations in FDG distribution within organs [20]. The liver, known for its significant role in glucose homeostasis, is a critical player in this context. FDG PET, therefore, serves as valuable supplementary information in the cancer detection process.

Hepatic enzymes, including AST, ALP, TP, bilirubin, and albumin, constitute routine hematological tests for assessing liver function (LFT); usually regulated by the liver, these enzymes are released into the bloodstream when the organ is injured or fails to control their secretion adequately, resulting in elevated enzyme levels in the host's body. In the context of cancer detection, heightened levels of liver enzymes may indicate liver damage or injury, potentially associated with various types of cancer [21]. However, it is important to emphasize that liver enzyme testing is highly sensitive but lacks specificity for various reasons. Liver enzyme polymorphisms present in diverse ethnic groups can lead to varying degrees of metabolism for different metabolites. Understanding these variations is important for accurate diagnosis and treatment, as what is considered a normal range of enzyme levels in one population might differ in another. Elevated enzyme levels may suggest liver damage or an inability to regulate enzyme levels, but they alone cannot be utilized for making a cancer diagnosis. Accurate determination of malignancy requires additional assessments and correlation with other clinical indicators, such as imaging studies like PET-CT.

PET/CT, combining functional and anatomical information, finds application in the initial staging and response assessment of breast cancer, lung cancer, Hodgkin, and non-Hodgkin lymphoma [22]. However, more studies are needed to explore the relationship between malignancy and enzyme levels. While PET/CT imaging plays a pivotal role in cancer detection and management, its accuracy can be influenced by serum enzyme levels. Researchers have even explored the predictive value of 2-[18F]FDG uptake in colorectal adenocarcinoma liver metastases (CLM) with regard to the KRAS mutational status [23]. The relationship between serum liver enzyme levels (SGOT/SGPT) and the liver's standard uptake values (SUV) on FDG-PET is complex and can be influenced by various factors related to liver function, disease, drug effects, metabolic status, and even the genetic makeup of an individual. This suggests that elevated liver enzyme levels may contribute to increased FDG uptake in the liver, potentially reducing the diagnostic sensitivity of FDG-PET for detecting cancer or infectious lesions. Consequently, a thorough evaluation of the liver on FDG-PET and correlation with other clinical manifestations become crucial in patients with elevated liver enzyme levels to prevent false-negative findings.

This study is distinctive in its inclusion of patients of Indian origin with diverse cancer types from a single facility, aiming to correlate liver serum enzyme markers with SUVmax. Such an investigation utilizing FDG-PET/CT across various tumor types has yet to be undertaken. We envisage that these results will hold particular relevance across different malignancies. The integration of FDG-PET/CT with serum enzyme levels has the potential to assist in identifying lesions suitable for localized radiotherapy.

In this research, we observed a positive correlation between ALP and SUVmax values in breast cancer, Hodgkin and non-Hodgkin lymphoma, and a negative correlation in lung cancer. Regarding albumin and SUVmax, a positive correlation was noted in lung cancer and Hodgkin lymphoma, while a negative correlation was observed in breast cancer and non-Hodgkin lymphoma. Total protein (TP) exhibited a positive relationship with SUVmax in lung cancer and Hodgkin lymphoma but a negative correlation in breast cancer and non-Hodgkin lymphoma. The ALT-to-AST ratio (ALT/AST) showed a positive correlation in lung cancer and Hodgkin lymphoma but a negative correlation in breast cancer and non-Hodgkin lymphoma. ALT values exhibited a positive correlation in lung cancer and non-Hodgkin lymphoma and a negative correlation in Hodgkin lymphoma.

Our study is limited by its retrospective design and the relatively small sample size. Nevertheless, considering the diversity of cancers and the limited research on the topic, our findings may hold significance. It is crucial to note that various statistical methodologies could be applied. However, in alignment with methods employed in previously published literature, we chose to assess the correlation between PET-derived parameters and liver serum enzymes using Pearson's coefficient and regression analysis.

## Conclusions

In summary, our research aids a fresh inclusion in early malignancy detection by analyzing liver serum enzyme levels (ALP, ALT, albumin, and TP) and tumor metabolic parameters. This suggests that the tumor metabolic parameter (SUVmax) can predict patient prognosis when correlated with certain liver serum enzyme levels. However, more detailed studies are needed to comprehensively understand the combined impact of these factors in tumor growth detection using PET/CT scans, offering crucial insights for improved therapeutic approaches in cancer treatment.
